# Low-Cost Nested-MIMO Array for Large-Scale Wireless Sensor Applications

**DOI:** 10.3390/s17051105

**Published:** 2017-05-12

**Authors:** Duo Zhang, Wen Wu, Dagang Fang, Wenqin Wang, Can Cui

**Affiliations:** 1 Ministerial Key Laboratory of JGMT, School of Electronic Engineering and Optoelectronic Technology, Nanjing University of Science and Technology, Nanjing 210094, China; wuwen@mail.njust.edu.cn (W.W.); fangdg@mail.njust.edu.cn (D.F); cuican100@163.com (C.C.); 2 School of Communication and Information Engineering, University of Electronic Science and Technology of China, Chengdu 611731, China; wqwang@uestc.edu.cn

**Keywords:** nested array, single receiver channel, time-sequence-phase-weighting (TSPW), multiple-input multiple-output (MIMO), degrees of freedom (DOF), large-scale array

## Abstract

In modern communication and radar applications, large-scale sensor arrays have increasingly been used to improve the performance of a system. However, the hardware cost and circuit power consumption scale linearly with the number of sensors, which makes the whole system expensive and power-hungry. This paper presents a low-cost nested multiple-input multiple-output (MIMO) array, which is capable of providing $O(2N^{2})$ degrees of freedom (DOF) with O(N) physical sensors. The sensor locations of the proposed array have closed-form expressions. Thus, the aperture size and number of DOF can be predicted as a function of the total number of sensors. Additionally, with the help of time-sequence-phase-weighting (TSPW) technology, only one receiver channel is required for sampling the signals received by all of the sensors, which is conducive to reducing the hardware cost and power consumption. Numerical simulation results demonstrate the effectiveness and superiority of the proposed array.

## 1. Introduction

The multiple-input multiple-output (MIMO) array has gained considerable attention recently since it is a flexible technique and can provide significant system performance improvement [1,2,3,4,5]. It forms a virtual array to efficiently extend the effective aperture and increase the degrees of freedom (DOF), which bring many benefits, such as spatial resolution enhancement [6], improved interference rejection capability [7] and excellent parameter identifiability [8]. The signal processing of the MIMO array is based on the amplitude and phase information received by each antenna element. To obtain the information, the general approach is to connect each array element with an independent receiver channel [9], which requires the number of receivers to be equal to that of the sensors. Since the receiver is one of the most expensive parts in the whole system, this requirement inevitably makes the system become complex, bulky and costly [10]. The problem is more serious in large-scale sensor arrays.

Over the last several years, many solutions have been developed to handle the challenge. One of the feasible solutions is the single receiver channel array based on the time-sequence-phase-weighting (TSPW) technology [11,12]. The single channel TSPW (SC-TSPW) array exchanges the sampling time for hardware cost savings. It weights and combines the signals of different sensors with orthogonal codes and decomposes them at the baseband by employing the same codes. Only one receiver channel is required for sampling. The basic principle of obtaining the original signals with one receiver channel is similar to that of code division multiple access (CDMA). The mentioned single channel array in the following paper will refer to the SC-TSPW array if there is no special instruction.

The modern communication and radar applications, i.e., 5th generation wireless systems (5G) [13], underwater sonar system [14] and personal radar [15], usually exploit large-scale sensor arrays to sample the spatial signals to improve the performance of the system. For a single channel array with *N* physical sensors, the sampling time for obtaining enough information to recover the original array signal is O(N). Therefore, although this type of array can effectively save the cost of the whole system, it is still a challenge to employ the single channel array in large-scale sensor applications because it will spend too much time on sampling. The larger the sensor scale is, the more serious the real-time processing problem will be.

In order to effectively increase the DOF, the nested array based on the concept of the Khatri–Rao (KR) product has been proposed in [16]. By producing a difference co-array (DCA), it is possible to obtain $O(N^{2})$ DOF from only O(N) physical sensors. Meanwhile, unlike the minimum redundancy arrays (MRAs) [17] and the minimum hole arrays (MHAs) [18], which need computer searching to find the positions of sensors, it has a simple closed-form expression for the array geometry, and there are no holes on the aperture of the corresponding DCA [19]. However, it mainly focuses on the applications of passive scenarios.

In this paper, a new array geometry named the single channel nested-MIMO array is introduced. The proposed array is formed by the fusion of the MIMO array, nested array and single channel array to produce a cost-effective and DOF-enhanced sensor array. It has a closed-form expression for the positions of transmit and receive arrays; thus, the aperture size and the number of DOF can be predicted. It will be demonstrated that by exploiting the proposed array, it is possible to obtain $O(2N^{2})$ DOF with only O(N) physical sensors. Additionally, compared with the conventional single channel array, the required number of receive sensors can be reduced for achieving a specified DOF.

The paper is organized as follows. Section 2 gives an overview of the related works to our research. In Section 3, the conventional single channel TSPW array, multi-channel MIMO array and nested array are briefly introduced. The algorithms for constructing the single channel nested-MIMO array and obtaining the original array signals through the single channel structure are proposed in Section 4. Simulation results, which show the effectiveness of the proposed array, are reported in Section 5, followed by conclusions drawn in Section 6.

## 2. Related Work

The single channel nested-MIMO array is based on the theory of the SC-TSPW array, the nested array and the MIMO array. The low-cost feature of the proposed array is inherited from the SC-TSPW array. In the previous studies on the conventional SC-TSPW array, the main focuses are all about the receive antenna design and the signal recovery method [11,20,21]. The system is considered to be single input multiple output (SIMO) by default. The number of receive sensors should be increased to enhance the DOF. The DOF grows linearly with the number of receive sensors.

Many of the researchers have already done much excellent work to enhance the degrees of freedom of the sensor array. An array named the nested MIMO array has been proposed in [22] for DOA estimation purpose. The co-array of the proposed array has been used to increase the DOF. However, the array geometry is the same as that of a normal MIMO array. Thus, the design complexity is the same as that of the standard MIMO array. In [23], another nested MIMO array based on the array construction methods of [24] has been proposed to increase the DOF. Additionally, the spatial sparsity of the array signal has been exploited for DOA estimation in this work. The minor defect is that the positions of the sensors are obtained by computer exhaustive search, which is difficult to be realized for large-scale wireless sensor applications. In [25], a nested minimum redundancy array (NMRA) has been proposed. It has a closed-form expression of the sensor locations, and the DOF has been significantly increased. The shortcoming of this array is that not all NMRA geometries with any number of sensors can be obtained since it relies on the structures of known MRAs. An array composed of phased-MIMO and nested-array has been proposed in [26] for increasing the DOF. The receiving array is a nested array. The proposed work benefits from both the nested array and the phased-MIMO array. However, only the receive array exploits the nested array structure, and the total number of sensors is still large.

To the best of our knowledge, the transmit antenna part has never been added into the SC-TSPW array before this study. To bridge this gap, the work tries to introduce the transmit sensor array to the SC-TSPW array and use the theories of MIMO and the nested array to enhance the degree of freedoms while maintaining the low-cost characteristic of the SC-TSPW array. The main contributions of this paper can be summarized as follows:

- The transmit antenna array is introduced into the conventional SC-TSPW array. Different waveforms transmitted by different antennas are exploited. The aperture size of the corresponding virtual array can be effectively extended. Specifically, since only one set of receiving equipment is required, the hardware cost is reduced compared with that of the frequently-used multichannel system. This low-cost feature is useful for those cost-sensitive applications, such as low-cost personal mobile radar [27,28], next generation wireless systems [29], millimeter-wave automotive radar [30], and so on.
- The work compromises the merits of the nested array and the MIMO array. The sensor locations of the proposed array have closed-form expressions. We prove with mathematic analysis that the virtual array of the proposed single channel nested-MIMO array can fully cover a parent nested array. The aperture size and number of DOF can be predicted as a function of the total number of sensors. Additionally, for a given number of DOF, the number of total sensors is relatively small.
- The corresponding signal recovery method of the single channel nested-MIMO array is proposed. Unlike the recovery method, which uses the single channel samplings that contain only one kind of waveform, the proposed signal recovery method uses the single channel samplings, which contains different orthogonal waveforms, to form a virtual array with a large aperture. For a given number of DOF, it requires less processing time than that of the conventional SC-TSPW array.

## 3. Preliminaries

### 3.1. Single-Channel TSPW Array

**Figure 1:**
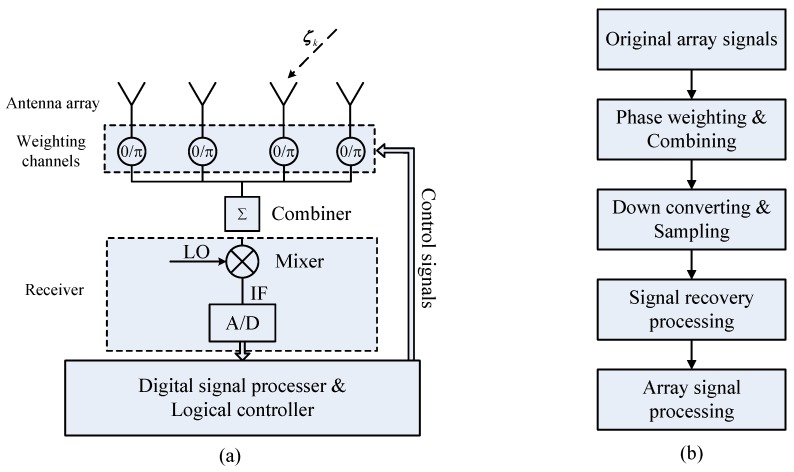
(**a**) Structure of a conventional SC-TSPW sensor array. The symbols LO, IF, A/D and $\zeta_{k}$ are the local oscillator, the intermediate frequency, the analog-to-digital converter and the radar reflection coefficient of the *k*-th target, respectively. (**b**) Signal processing block diagram of the single channel system.

The system structure and the signal processing flowchart of a conventional SC-TSPW array are shown in Figure 1.

The original spatial array signals $X={\left[x_{1},\ldots ,x_{N}\right]}^{T}$ are sampled with a fully filled sensor array. Each sensor is connected with an independent digital controlled 0/π phase shifter, which produces a weighting vector $W=[w_{i1},\ldots ,w_{iN}]$ composed of one and −1. For an array with *N* elements, these shifters change state *N* times to form an orthogonal Walsh–Hadamard matrix W and produce the single channel outputs $Y={\left[y_{1},\ldots ,y_{N}\right]}^{T}$, which can be written as [31]:

(1) $$Y=\mathrm{WX}+E=\left[\begin{array}{ccc} w_{11} & \cdots & w_{1N}\\ \vdots & \ddots & \vdots \\ w_{N1} & \cdots & w_{\mathrm{NN}} \end{array}\right]\left[\begin{array}{c} x_{1}\\ \vdots \\ x_{N} \end{array}\right]+\left[\begin{array}{c} e_{1}\\ \vdots \\ e_{N} \end{array}\right]$$

where $E={\left[e_{1},e_{2},\ldots ,e_{N}\right]}^{T}$ is the noise term.

With only one RF receive channel, the single channel outputs Y are down converted, sampled and stored temporally. With the signal recovery algorithm, the original array signal can be recovered in the digital domain by:

(2) $$\overset{˘}{X}=W^{-1}Y=W^{-1}\left(\mathrm{WX}+E\right)=X+\frac{1}{N}W^{T}E$$

where $W^{-1}=\frac{1}{N}W^{T}$ is the inverse matrix of the Walsh–Hadamard matrix and $W^{T}W=NI_{N}$ is the property of Walsh–Hadamard matrix.

**Figure 2:**
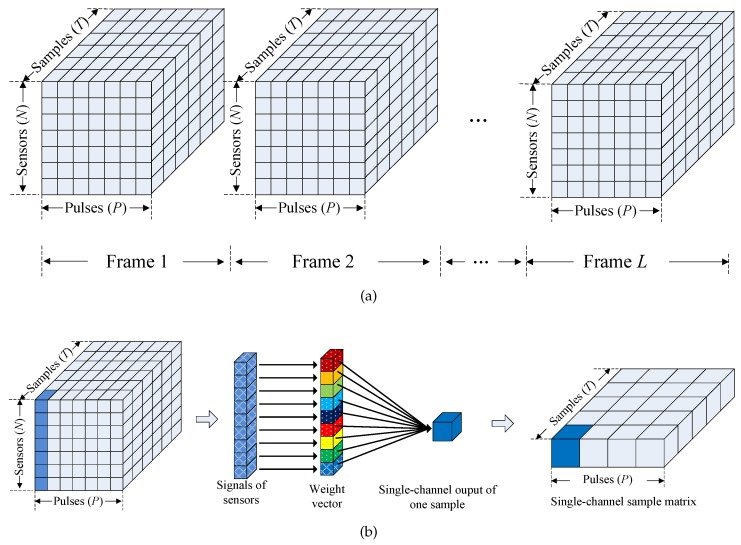
(**a**) Array signal storage model for a pulse radar with the sensor array. (**b**) Single channel sampling procedure in the view of datacube.

According to [32], the echo signal of pulse radar with the sensor array after being converted into the baseband can be considered as stored in a three-dimensional structure, called a datacube, as shown in Figure 2a. In the view of the datacube, the procedure of the single channel sampling is illustrated in Figure 2b. The original datacube degenerates into a single channel sample matrix after the single channel sampling. It can be recovered when the single channel sample matrix contains enough information. For an SC-TSPW array with *N* DOF (*N* is a power of two), *N* pulses must be taken to provide enough information to form the single channel data matrix. Thus, the sampling time of SC-TSPW will increase linearly with the number of sensors, which produces serious real-time processing problems and leads it to become unsuitable for large-scale measurement arrays.

### 3.2. Multi-Channel MIMO Array

Unlike the phased-array radar, the standard MIMO radar exploits the waveforms by transmitting different waveforms at different antennas. Consider a multi-channel MIMO system with a transmit array with *M* antenna sensors and a receive array with *N* antenna sensors. There are mainly two types of MIMO antennas: one is the widely separated antenna array [33], and the other is the co-located antenna array [34]. This work addresses the co-located antenna array, for which both transmit and receive arrays are closely located. The transmitted signal of the *m*-th sensor can be expressed in low-pass equivalent form as:

(3) $$s_{m}\left(t\right)=\sqrt{\frac{E}{M}}\phi_{m}\left(t\right),\;m=1,\ldots ,M,\;0\leq t\leq T_{p}$$

where *E* is the total transmitted energy within one radar pulse, $T_{p}$ is the pulse width and $\phi_{m}\left(t\right)$ is the energy normalized orthogonal waveform, which satisfies $\int_{T_{p}}{\left|\phi_{m}\left(t\right)\right|}^{2}dt=1$. In this paper, the set of frequency spread signals proposed in [35] is adopted as $\phi_{m}\left(t\right)$. It can be described as:

(4) $$\phi_{m}\left(t\right)=\left\{\begin{array}{cc} \frac{1}{\sqrt{T_{p}}}\exp\left(j2\pi m\Delta ft\right), & 0<t<T_{p}\\ 0, & \mathrm{otherwise} \end{array}\right.$$

where m=1,…,M. It is shown in [35] that these signals satisfy $\int_{T_{p}}\phi_{m}\left(t\right)\phi_{m}\left(t\right)dt=1,m=n$ and $\int_{T_{p}}\phi_{m}\left(t\right)\phi_{n}^{*}\left(t\right)dt\approx 0,m\neq n$, if the frequency difference $\Delta f\gg 1/T_{p}$.

Assume that the number of targets is *K*. The far-field baseband signal received by the sensor array of the *p*-th pulse of the *l*-th frame is [36]:

(5) $$x_{p,l}\left(t\right)=\sqrt{\frac{E}{M}}\sum_{k=1}^{K}\zeta_{k,l}b\left(\theta_{k}\right)a^{T}\left(\theta_{k}\right)\phi \left(t\right)+z_{p,l}\left(t\right)$$

where *t*, θ and ζ are the fast time index, i.e., the snapshot number, spatial angle and the reflection coefficient with variance $\sigma_{k}^{2}$, respectively, $\phi (t)={\left[\phi_{1}\left(t\right),\ldots ,\phi_{M}\left(t\right)\right]}^{T}$ is the waveform vector, ${\left(\cdot \right)}^{T}$ denotes the transpose $z_{p,l}\left(t\right)$ is N×1 zero-mean white Gaussian noise term and

(6) $$\begin{array}{c} a\left(\theta_{k}\right)={\left[1,e^{-j\frac{2\pi }{\lambda }\sin\left(\theta_{k}\right)d_{s}},\ldots ,e^{-j\frac{2\pi }{\lambda }\sin\left(\theta_{k}\right)\left(M-1\right)d_{s}}\right]}^{T}\\ b\left(\theta_{k}\right)={\left[1,e^{-j\frac{2\pi }{\lambda }\sin\left(\theta_{k}\right)d_{r}},\ldots ,e^{-j\frac{2\pi }{\lambda }\sin\left(\theta_{k}\right)\left(N-1\right)d_{r}}\right]}^{T} \end{array}$$

are the steering vectors of transmit and receive array, respectively, λ is the wavelength and $d_{s}$ and $d_{r}$ are the spacing of adjacent elements of transmit and receive array, respectively. Note that the radar cross-section of the target is assumed to be constant from pulse to pulse, but varies independently from frame to frame with variance $\sigma_{k}^{2}$, i.e., it obeys the Swerling Case I target model [37].

Exploiting the orthogonality property, the received signal can be decomposed by *M* matched filters. After matched filtering, the MIMO signal vector of the *p*-th pulse can be obtained, which is given as follows:

(7) $$X_{p,l}=\sqrt{\frac{E}{M}}\sum_{k=1}^{K}\zeta_{k,l}b\left(\theta_{k}\right)a^{T}\left(\theta_{k}\right)+{\tilde{z}}_{p,l}$$

where ${\tilde{z}}_{p,l}$ is the noise vector. By stacking the signal $X_{p,l}$ in one column, the virtual data vector can be obtained. The dimensionality of the data vector can reflect the number of DOF of the MIMO array. In particular, if $d_{s}=Nd_{r}$ is chosen to form an MIMO array, the corresponding virtual array is a uniform linear array (ULA), and the number of DOF is MN, which is just the number of sensors of the virtual array [16].

### 3.3. Nested Array

The nested array is a concatenation of two uniform linear arrays (ULAs) [38]: the inner and outer arrays, where the inner ULA has $N_{1}$ sensors with spacing $d_{1}$ and the outer ULA has $N_{2}$ sensors with spacing $d_{2}$. Although there are many types of nested arrays, we only focus on the two-level nested array because the difference co-array (DCA) produced by the two-level nested array has no holes on the aperture. The DCA of the two-level nested array is a fully-filled ULA with $2N_{2}\left(N_{1}+1\right)-1$ virtual sensors. Thus, the degrees of freedom (DOF) that the two-level nested array can provide is $2N_{2}\left(N_{1}+1\right)-1$. It should be noted that the minimum redundancy MIMO proposed in [24] can also form the DCA and obtain more DOF than those of the nested array. However, only by computer searching can the positions of the sensors of the minimum redundancy MIMO be obtained. In the following context, the nested array will refer to the two-level nested array without additional instructions.

To obtain the signals of the associated DCA, it is necessary to compute the source covariance matrix R. In practice, the covariance matrix can be estimated using the samples of *L* frames [39] as $R=\left(1/L\right)\sum_{l=0}^{L-1}X\left(l\right)X^{H}\left(l\right)$, where ${\left(\cdot \right)}^{H}$ is the conjugation transpose operator. The signals of DCA can be written as:

(8) $$\hat{X}=\mathrm{vec}\left(R\right)=(B^{*}⊙B)\sigma +{\tilde{e}}_{n}$$

where $\sigma ={\left[\sigma_{1}^{2},\ldots ,\sigma_{K}^{2}\right]}^{T}$, $B=[b\left(\theta_{1}\right),\ldots ,b\left(\theta_{K}\right)]$, ${\tilde{e}}_{n}$ is noise vector and ⊙ denotes the Khatri–Rao (KR) product of two matrices.

## 4. Single-Channel Nested-MIMO Array

### 4.1. Array Geometry of Single-Channel Nested-MIMO Array

**Figure 3:**
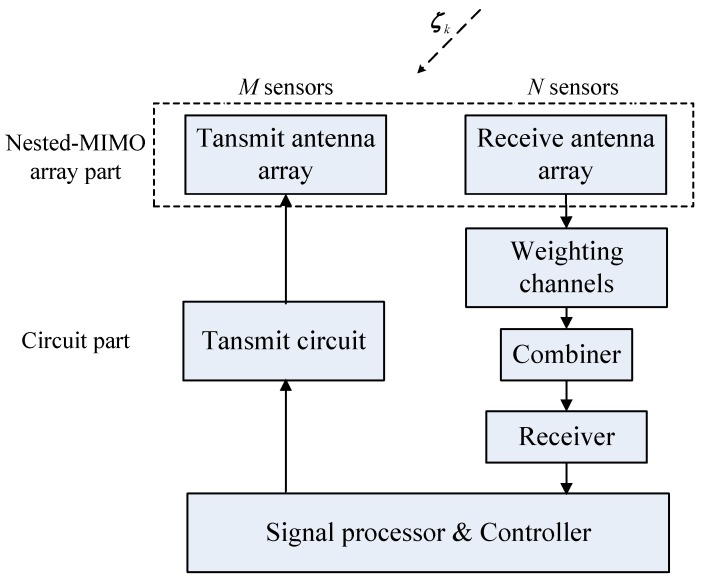
Structure of the single channel nested-MIMO array.

It is shown that the two-level nested array has an optimized distribution of sensors to maximize the total DOF [16]. Therefore, in this section, we consider constructing an MIMO array, which makes the corresponding virtual array contain the optimized two-level nested array while maintaining the number of total sensors as few as possible. Additionally, the receive array will exploit the single channel structure to reduce the cost and power consumption of the system. The structure diagram of the single channel nested-MIMO array is illustrated in Figure 3.

Assume the transmit and receive sensor arrays are parallel and co-located. Their positions are all from a virtual uniform grid, where the minimum grid spacing is chosen as d=λ/2 to avoid spatial aliasing. Denote $P_{t}=\left\{\vec{p_{T_{i}}}|i=1,\ldots ,M\right\}$ and $P_{r}=\left\{\vec{p_{R_{j}}}|j=1,\ldots ,N\right\}$ as the set of indexes of the positions of transmit and receive elements, respectively, where $\vec{p_{T_{i}}}$ and $\vec{p_{R_{j}}}$ are the positions of the *i*-th transmit and the *j*-th receive sensors, respectively. The single channel nested-MIMO arrays can be constructed according to the following array geometry:

**Definition** **1.** *(Second-order single channel nested-MIMO arrays): Assume $N_{1}$ and $N_{2}$ are integers satisfying $N_{1}\geq 1$ and $N_{2}\geq 2$. The single channel nested-MIMO arrays are specified by the integer sets $P_{t}$ and $P_{r}$, defined by:*

(9) $$\begin{array}{c} P_{t}=X_{1}\cup X_{2}\\ P_{r}=Y_{1} \end{array}$$

*where:*

(10) $$\begin{array}{c} X_{1}=\left\{1+iB|0\leq i\leq 1\right\},\\ Y_{1}=\left\{i|1\leq i\leq B\right\},\\ X_{2}=\left\{iA-B+1|2\leq i\leq N_{2}\right\}. \end{array}$$

*The parameters A and B are defined as:*

(11) $$A=N_{1}+1,B=\left\lceil \frac{N_{1}+1}{2}\right\rceil$$

*where ⌈·⌉ denotes the convention of rounding towards positive infinity.*

According to the Definition 1, we have:

**Lemma** **1.** When the number of sensors of the parent nested array N≥3, the virtual array of the second-order single channel nested-MIMO array has the same aperture size as that of the parent nested array.

**Proof:**  The virtual array is the convolution of the transmit and receive arrays [40]. The sensor positions of the virtual array are given by the set $P_{V}=\left\{\vec{p_{T_{i}}}+\vec{p_{R_{j}}},\;i=1,\ldots ,N,j=1,\ldots ,M\right\}$. Assume the parent nested array has $N=N_{1}+N_{2}\;\left(N\geq 3\right)$ sensors. Then, the aperture size of the nested array is $AS_{nested}=\left(N_{2}\left(N_{1}+1\right)-1\right)d$ [16]. The aperture size of the proposed array is:

(12) $$\begin{array}{cc} \bar{AS} & =(\max\left\{P_{t}\right\}+\max\left\{P_{r}\right\}-2)d\\ & =(\max\left\{X_{2}\right\}+\max\left\{Y_{1}\right\}-2)d\\ & =(N_{2}A-B+1+B-2)d\\ & =(N_{2}\left(N_{1}+1\right)-1)d \end{array}$$

Thus, $AS_{nested}=\bar{AS}$ completes the proof. Q.E.D.  ☐

**Theorem** **1.** For the nested array ($N_{Total}\geq 3$), there exists at least one single channel nested-MIMO array, such that the associated virtual array covers the parent nested array.

**Proof:**  Because the proposed array has the same aperture size as that of the parent nested array (Lemma 1), the statement of Theorem 1 is equivalent to the following argument: if the position set $P_{nested}$ of the parent nested array is a subset of $P_{V}$, then the parent nested array is covered by the virtual array of the single channel nested-MIMO array. The position set $P_{nested}$ can be considered as the union of two sets: the inner set $P_{inner}$ and the outer set $P_{outer}$, where:

(13) $$\begin{array}{ccc} P_{inner} & = & \left\{i|1\leq i\leq N_{1}\right\} \end{array}$$

(14) $$\begin{array}{ccc} \;P_{outer} & = & \left\{i\left(N_{1}+1\right)|1\leq i\leq N_{2}\right\} \end{array}$$

First, we consider the situation when $N_{1}$ is even. Let $N_{1}=2j,\;j\in N^{+}$. Then $A=N_{1}+1=2j+1$, $B=\left\lceil \frac{N_{1}+1}{2}\right\rceil =\left\lceil \frac{2j+1}{2}\right\rceil =j+1$. According to Definition 1, we have:

(15) $$\begin{array}{c} X_{1}=\left\{1,j+2\right\}\\ Y_{1}=\left\{1,\ldots ,j,j+1\right\} \end{array}$$

According to [16], for the optimal distribution, $N_{2}$ should be 2j or 2j+1. The proofs of these two case are nearly the same. Thus, we only discuss the situation of the former one for explanation purposes. When $N_{2}=2j,\;j\in N^{+}$,

(16) $$\begin{array}{cc} X_{2} & =\{2A-B+1,3A-B+1,\ldots ,N_{2}A-B+1\},\\ & =\{2(2j+1)-(j+1)+1,3(2j+1)-(j+1)+1,\ldots ,2j(2j+1)-(j+1)+1\},\\ & =\{3j+2,5j+3,\ldots ,4j^{2}+j\}. \end{array}$$

Thus, the position set of the virtual array $P_{V}$ is:

(17) $$\begin{array}{cc} P_{V}= & \left\{1,\ldots ,j,j+1\}\cup \{j+2,\ldots ,2j,2j+1,2j+2\right\}\\ & \cup \left\{3j+2,\ldots ,4j+1,4j+2\right\}\cup \ldots \cup \left\{4j^{2}+j,\ldots ,4j^{2}+2j-1,4j^{2}+2j\right\} \end{array}$$

Since the position set of the nested array is:

(18) $$\begin{array}{cc} P_{nested} & =P_{inner}\cup P_{outer}\\ & =\{1,\ldots ,2j,2j+1,4j+2,\ldots ,4j^{2}+2j\}, \end{array}$$

it is clear that each element in the set $P_{nested}$ is contained in the set $P_{V}$. Thus, $P_{nested}$ is a subset of $P_{V}$, which is denoted as $P_{nested}\subseteq P_{V}$. The proof is completed. When $N_{1}$ is odd, the proof is quite similar to the above one, and we do not repeat it. Q.E.D.  ☐

**Corollary** **1.** *If the parent nested array contains $N=N_{1}+N_{2}\geq 3$ sensors, the total number $N_{Total}$ of physical sensors and the number $N_{s}$ of single channel receive sensors of the second-order single channel nested-MIMO arrays are:*

(19) $$\left(N_{Total},N_{s}\right)=\left\{\begin{array}{c} (\frac{N_{1}+2N_{2}+4}{2},\frac{N_{1}+2}{2}),\;\mathrm{if}\;N_{1}\;\mathrm{is}\;\mathrm{even}\\ (\frac{N_{1}+2N_{2}+3}{2},\frac{N_{1}+1}{2}),\;\mathrm{if}\;N_{1}\;\mathrm{is}\;\mathrm{odd} \end{array}\right.$$

**Proof:**  The number of total sensors $N_{Total}$ is equal to the sum of the numbers of elements of three sets $X_{1}$, $X_{2}$ and $Y_{1}$, and the number of single channel receive sensors $N_{s}$ is equal to the number of elements of $Y_{1}$ in Equation (10), which is also equal to the value of *B*. If $N_{1}$ is even, then $B=\frac{N_{1}}{2}+1$ and:

(20) $$\begin{array}{cc} N_{Total} & =2+\frac{N_{1}}{2}+1+N_{2}-1\\ & =\frac{N_{1}}{2}+N_{2}+2,\\ N_{s} & =\frac{N_{1}}{2}+1. \end{array}$$

If $N_{1}$ is odd, then $B=\frac{N_{1}}{2}+0.5$ and:

(21) $$\begin{array}{cc} N_{Total} & =2+\frac{N_{1}}{2}+0.5+N_{2}-1\\ & =\frac{N_{1}}{2}+N_{2}+1.5,\\ N_{s} & =\frac{N_{1}}{2}+0.5. \end{array}$$

Q.E.D.  ☐

**Figure 4:**
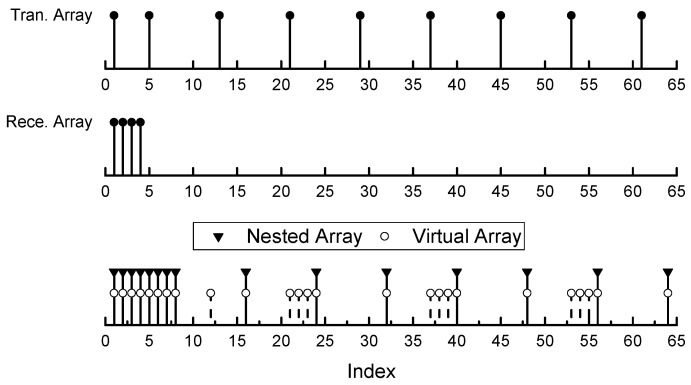
Scheme of a single channel nested-MIMO array with 13 sensors, which covers a nested array with 15 sensors. The position sets of the single channel nested-MIMO array are $P_{t}=\left\{1,5,13,21,29,37,45,53,61\right\}$ and $P_{r}=\left\{1,2,3,4\right\}$, respectively.

With Definition 1, it is easy to construct a single channel nested-MIMO array whose virtual array totally contains the target nested array. Additionally, the positions of the sensors have the exact closed-form expression. Figure 4 shows an example of a single channel nested-MIMO array with 13 physical sensors, which covers a parent nested array with 15 sensors. The set of positions of the parent nested array is $P_{nested}=\{1,2,3,4,5,6,7,8,16,24,32,40,48,$ 56,64}, and the set of virtual arrays of the single channel nested-MIMO array is $P_{V}=\{1,2,3,4,5,6,7,8,13,16,21,22,23,24,$ 32,37,38,39,40,48,53,54,55,56,64}.

Although the single channel nested-MIMO array constructed by Definition 1 can correctly contain the required nested array, it can be seen that there are many unused virtual elements in the virtual array, which increases the redundancy. The high redundancy requires more physical sensors to form the array, which increases the cost and power consumption. To further reduce the total number of sensors, we loosen the restriction on the minimum number of total sensors and develop an optimized array based on Definition 1.

**Definition** **2.** *(Optimized second-order single channel nested-MIMO arrays): Assume $N_{1}$ and $N_{2}$ are integers satisfying $N_{1}\geq 3$ and $N_{2}\geq 4$. The optimized second-order single channel nested-MIMO arrays are specified by the integer sets $P_{s}$ and $P_{r}$, defined by:*

(22) $$\begin{array}{c} P_{t}=X_{1}\cup X_{2}\cup X_{3}\\ P_{s}=Y_{1}\cup Y_{2} \end{array}$$

*where:*

(23) $$\begin{array}{c} X_{1}=\left\{1+iB|0\leq i\leq 1\right\},Y_{1}=\left\{i|1\leq i\leq B\right\},\\ X_{2}=\left\{\begin{array}{cc} \;\;\;\;\{\emptyset \}, & \;\mathrm{if}\;N_{2}\;\mathrm{is}\;\mathrm{even}\\ \;\;\;\;\{3A-B+1\}, & \;\mathrm{if}\;N_{2}\;\mathrm{is}\;\mathrm{odd}\;\mathrm{and}\;N_{1}\;\mathrm{is}\;\mathrm{even}\\ \;\;\;\;\{A+3B+2\}, & \;\mathrm{if}\;N_{2}\;\mathrm{is}\;\mathrm{odd}\;\mathrm{and}\;N_{1}\;\mathrm{is}\;\mathrm{odd} \end{array}\right.\\ Y_{2}=\left\{\begin{array}{cc} \;\;\;\;\{2A-B\}, & \;\mathrm{if}\;N_{2}\;\mathrm{is}\;\mathrm{even}\\ \;\;\;\;\{iA|1\leq i\leq 2\}, & \;\mathrm{if}\;N_{2}\;\mathrm{is}\;\mathrm{odd}\;\mathrm{and}\;N_{1}\;\mathrm{is}\;\mathrm{even}\\ \;\;\;\;\{2A-B\}, & \;\mathrm{if}\;N_{2}\;\mathrm{is}\;\mathrm{odd}\;\mathrm{and}\;N_{1}\;\mathrm{is}\;\mathrm{odd} \end{array}\right.\\ X_{3}=\left\{\begin{array}{cc} \{2iA+B+1|1\leq i\leq C\}, & \;\mathrm{if}\;N_{2}\;\mathrm{is}\;\mathrm{even}\\ \{(2i+1)A+1|1\leq i\leq C\}, & \;\mathrm{if}\;N_{2}\;\mathrm{is}\;\mathrm{odd}\;\mathrm{and}\;N_{1}\;\mathrm{is}\;\mathrm{even}\\ \{(2i+1)A+B+1|1\leq i\leq C\}, & \;\mathrm{if}\;N_{2}\;\mathrm{is}\;\mathrm{odd}\;\mathrm{and}\;N_{1}\;\mathrm{is}\;\mathrm{odd} \end{array}\right. \end{array}$$

*The parameters A, B and C are defined as:*

(24) $$\left(A,B,C\right)=\left\{\begin{array}{cc} (N_{1}+1,\left\lceil \frac{N_{1}+1}{2}\right\rceil ,\frac{N_{2}-2}{2}), & \;\mathrm{if}\;N_{2}\;\mathrm{is}\;\mathrm{even}\\ (N_{1}+1,\left\lfloor \frac{N_{1}+1}{2}\right\rfloor ,\frac{N_{2}-3}{2}), & \;\mathrm{if}\;N_{2}\;\mathrm{is}\;\mathrm{odd} \end{array}\right.$$

*where ⌊·⌋ denotes the convention of rounding towards negative infinity and* ∅ *denotes the null set.*

**Lemma** **2.** When the number of sensors of the parent nested array N≥7, the aperture size of the virtual array of the optimized single channel nested-MIMO array is equal to that of the parent nested array.

**Proof:**  The aperture size of the nested array is $AS_{nested}=\left(N_{2}\left(N_{1}+1\right)-1\right)d$. Without loss of generality, we assume $N_{2}$ is even. Then, $A=N_{1}+1$, $C=\frac{N_{2}}{2}-1$. The aperture size of the optimized single channel nested-MIMO array, when N≥7, is:

(25) $$\begin{array}{cc} \bar{AS_{opt.}} & =(\max\left\{P_{t}\right\}+\max\left\{P_{r}\right\}-2)d\\ & =(\max\left\{X_{3}\right\}+\max\left\{Y_{2}\right\}-2)d\\ & =(2AC+B+1+2A-B-2)d\\ & =(2A(C+1)-1))d\\ & =(N_{2}\left(N_{1}+1\right)-1)d \end{array}$$

It is clear that $AS_{nested}=\bar{AS_{opt.}}$. The proof of the case when $N_{2}$ is odd is similar except that the value of $N_{1}$ should be taken into consideration in the proof. Q.E.D.  ☐

**Theorem** **2.** For the nested array (N≥7), there exists at least one optimized second-order single channel nested-MIMO array such that the associated virtual array fully covers the parent nested array.

**Proof:**  Because the optimized single channel nested-MIMO array has the same aperture size as that of the parent nested array, we need to prove that the position set $P_{nested}$ is a subset of $P_{V}$. Consider the situation when $N_{2}$ is odd and $N_{1}$ is even. Let $N_{2}=2j+3,\;N_{1}=2j+2,\;j\in N^{+}$. The inner and outer position sets of the nested array are:

(26) $$\begin{array}{ccc} P_{inner} & = & \{1,2,\ldots ,2j+2\}, \end{array}$$

(27) $$\begin{array}{ccc} \;P_{outer} & = & \{2j+3,4j+6,\ldots ,{\left(2j+3\right)}^{2}\}. \end{array}$$

The position set of the nested array is:

(28) $$\begin{array}{cc} P_{nested} & =P_{inner}\cup P_{outer}\\ & =\{1,\ldots ,2j+2,2j+3,4j+6,\ldots ,4j^{2}+12j+9\}. \end{array}$$

According to Equation (24), A=2j+3,B=j+1,C=j. Substitute A,B,andC into Equation (23); we get:

(29) $$\begin{array}{cc} X_{1} & =\left\{1,j+2\right\},\;X_{2}=\left\{5j+9\right\},\\ X_{3} & =\{6j+10,10j+16,\ldots ,4j^{2}+8j+4\},\\ Y_{1} & =\left\{1,\ldots ,j+1\right\},\;Y_{2}=\left\{2j+3,4j+6\right\}. \end{array}$$

Then:

(30) $$\begin{array}{ccc} P_{t} & = & \{1,j+2,5j+9,6j+10,10j+16,\ldots ,4j^{2}+8j+4\}, \end{array}$$

(31) $$\begin{array}{ccc} P_{r} & = & \{1,\ldots ,j+1,2j+3,4j+6\}.\; \end{array}$$

The sensor position of the virtual array $P_{V}$ is listed in Table 1 as follows.

It can be verified that the upper-left data block of Table 1 is just the set $P_{inner}$, and the other elements of Table 1 contain the set $P_{outer}$ with redundancy. The proofs of the other cases are nearly the same as this one, and we will not repeat them. Q.E.D.  ☐

**Corollary** **2.** *If the parent nested array contains $N=N_{1}+N_{2}\geq 7$ sensors, the total number $N_{Total}$ of physical sensors and the number $N_{s}$ of single channel receiving sensors of the optimized second-order single channel nested-MIMO array are:*

(32) $$\left(N_{Total},N_{s}\right)=\left\{\begin{array}{cc} (\frac{N_{1}+N_{2}+6}{2},\frac{N_{1}+4}{2}), & \;\mathrm{if}\;N_{2}\;\mathrm{is}\;\mathrm{even}\;\mathrm{and}\;N_{1}\;\mathrm{is}\;\mathrm{even}\\ (\frac{N_{1}+N_{2}+5}{2},\frac{N_{1}+3}{2}), & \;\mathrm{if}\;N_{2}\;\mathrm{is}\;\mathrm{even}\;\mathrm{and}\;N_{1}\;\mathrm{is}\;\mathrm{odd}\\ (\frac{N_{1}+N_{2}+7}{2},\frac{N_{1}+4}{2}), & \;\mathrm{if}\;N_{2}\;\mathrm{is}\;\mathrm{odd}\;\mathrm{and}\;N_{1}\;\mathrm{is}\;\mathrm{even}\\ (\frac{N_{1}+N_{2}+6}{2},\frac{N_{1}+3}{2}), & \;\mathrm{if}\;N_{2}\;\mathrm{is}\;\mathrm{odd}\;\mathrm{and}\;N_{1}\;\mathrm{is}\;\mathrm{odd} \end{array}\right.$$

**Proof:**  The proof is nearly the same as the proof of corollary 1, and we will not repeat it here. Q.E.D.  ☐

**Figure 5:**
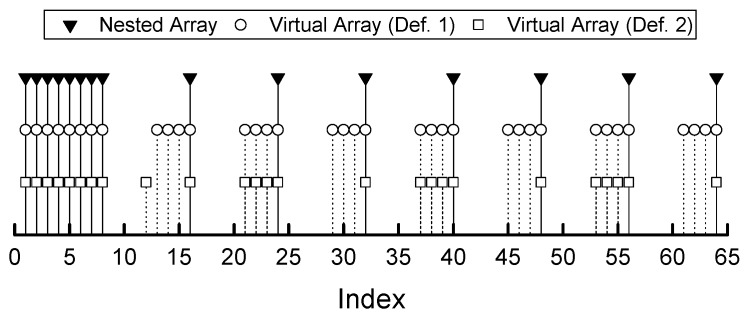
Comparison of the array geometry of the parent nested array and two virtual arrays. The virtual arrays are associated with the single channel nested-MIMO arrays that were generated by Definitions 1 and 2, respectively.

As an example, Figure 5 shows the virtual arrays produced by the single channel nested-MIMO arrays that constructed by Definition 1 and 2, respectively. The target nested array is the same as that in Figure 4. It can be seen from Figure 5 that Definition 2 can produce a satisfactory array with less physical sensors. The position indexes of the sensors generated by Definition 2 are $P_{s}=\left\{1,5,21,37,53\right\}$ and $P_{r}=\left\{1,2,3,4,12\right\}$, respectively. The number of total physical sensors is 10, which is less than that of the array produced by Definition 1.

Given a number of sensors $N_{Total}$, the designer usually is concerned about how many DOF the array can provide. According to Definitions 1 and 2, the number of DOF that the proposed array can provide is given by the following corollary:

**Corollary** **3.** *Given a number $N_{Total}\left(N_{Total}\geq 6\right)$ of physical sensors, the maximal number of DOF that the single channel nested-MIMO array can provide from the associated DCA is:*

(33) $$DOF_{nested-MIMO}=\left\{\begin{array}{cc} 2N_{Total}^{2}-8N_{Total}+7, & \;\mathrm{if}\;N_{Total}\;\mathrm{is}\;\mathrm{even}\\ 2N_{Total}^{2}-10N_{Total}+11, & \;\mathrm{if}\;N_{Total}\;\mathrm{is}\;\mathrm{odd} \end{array}\right.$$

**Proof:**  When $N_{Total}\geq 7$, the maximal number of DOF is provided by the optimized second-order single channel nested-MIMO arrays. Because the aperture size of the parent nested array of the optimized array is larger than that of the original nested-MIMO array, under the constraint of a fixed total number of sensors, for a parent nested-array with $N=N_{1}+N_{2}$ sensors, the number of DOF that it can provide is [16]:

(34) $$DOF_{nested}=\left\{\begin{array}{cc} \frac{N^{2}-2}{2}+N, & \;\mathrm{if}\;N\;\mathrm{is}\;\mathrm{even}\\ \frac{N^{2}-1}{2}+N, & \;\mathrm{if}\;N\;\mathrm{is}\;\mathrm{odd} \end{array}\right.$$

According to Equation (32), we will discuss it in four cases.

1. If $N_{1}$ is an odd integer, and $N_{2}$ is an even integer:
  In this case, the number of sensors of the parent nested-array *N* is an odd integer and the number of physical sensors of the nested-MIMO array $N_{Total}$ is an even integer. Then:
  (35) $$\begin{array}{cc} DOF(1) & =\frac{N^{2}-1}{2}+N\\ & =\frac{{\left(N_{1}+N_{2}\right)}^{2}-1}{2}+\left(N_{1}+N_{2}\right)\\ & =\frac{{\left(2N_{Total}-5\right)}^{2}-1}{2}+\left(2N_{Total}-5\right)\\ & =2N_{Total}^{2}-8N_{Total}+7 \end{array}$$
2. If both $N_{1}$ and $N_{2}$ are even integers:
  In this case, the number of sensors of the parent nested-array *N* is an even integer, and the number of physical sensors of the nested-MIMO array $N_{Total}$ is an odd integer. Then:
  (36) $$\begin{array}{cc} DOF(2) & =\frac{N^{2}-2}{2}+N\\ & =\frac{{\left(N_{1}+N_{2}\right)}^{2}-2}{2}+\left(N_{1}+N_{2}\right)\\ & =\frac{{\left(2N_{Total}-6\right)}^{2}-2}{2}+\left(2N_{Total}-6\right)\\ & =2N_{Total}^{2}-10N_{Total}+11 \end{array}$$
3. If $N_{1}$ is an even integer and $N_{2}$ is an odd integer:
  In this case, the number of sensors of the parent nested-array *N* is an odd integer, and the number of physical sensors of the nested-MIMO array $N_{Total}$ is an even integer. Then:
  (37) $$\begin{array}{cc} DOF(3) & =2N_{Total}^{2}-12N_{Total}+17 \end{array}$$
4. If both $N_{1}$ and $N_{2}$ are odd integers:
  In this case, the number of sensors of the parent nested-array *N* is an even integer and the number of physical sensors of the nested-MIMO array $N_{Total}$ is an even integer. Then:
  (38) $$\begin{array}{cc} DOF(4) & =2N_{Total}^{2}-10N_{Total}+11 \end{array}$$
  Thus, the maximal number of DOF that the nested-MIMO array can provide is:
  (39) $$\begin{array}{cc} DOF_{nested-MIMO} & =\max\{DOF(1),DOF(2),DOF(3),DOF(4)\}\\ & =\left\{\begin{array}{cc} 2N_{Total}^{2}-8N_{Total}+7, & \;\mathrm{if}\;N_{Total}\;\mathrm{is}\;\mathrm{even}\\ 2N_{Total}^{2}-10N_{Total}+11, & \;\mathrm{if}\;N_{Total}\;\mathrm{is}\;\mathrm{odd} \end{array}\right. \end{array}$$
  Q.E.D.  ☐

### 4.2. Signal Processing of Single Channel Nested-MIMO Array

**Figure 6:**
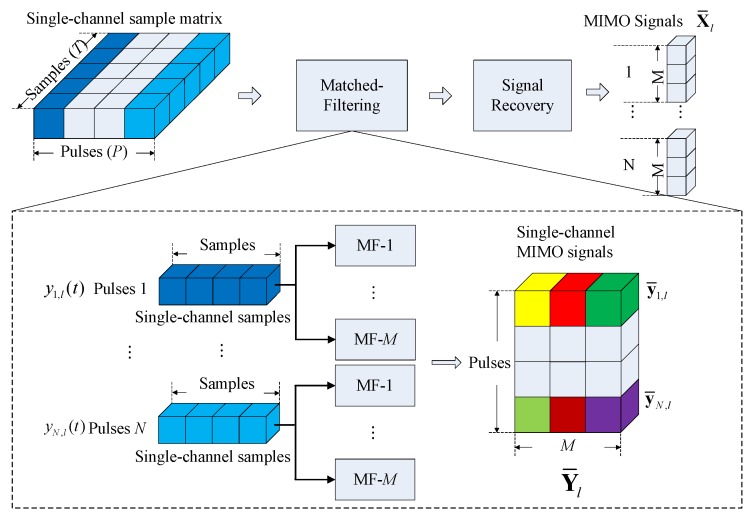
Signal processing diagram of the signal channel MIMO array.

Because the proposed array exploits the single RF receiving channel to sample the original array signals, the samplings cannot be directly used for subsequent array signal processing. The original array signals should first be recovered by using the single channel sampling data. The processing flow is shown in Figure 6. Since the signal processing methods of different frames are the same, we only discuss the procedure in one frame.

Specifically, before recovering the original signals from the single channel data matrix, waveform matched filters should be added. To reduce the requirement of the bandwidth of phase shifters, the same weighting vector is used in one pulse, and different weighting vectors are used for different pulses. Consider a single channel nested-MIMO array composed of a transmit array with *M* co-located sensors and a single channel receive array with *N* sensors. Following Equation (5), the single channel samplings of the *p*-th pulse in the *l*-th frame $y_{p,l}\left(t\right)$ can be expressed as:

(40) $$\begin{array}{cc} y_{p,l}\left(t\right) & =H_{p}^{T}x_{p,l}\left(t\right)\\ & =H_{p}^{T}\sum_{k=1}^{K}\zeta_{k,l}^{\prime }b\left(\theta_{k}\right)a_{m}^{T}\left(\theta_{k}\right)\phi \left(t\right)+H_{p}^{T}z_{p,l}\left(t\right) \end{array}$$

where $H_{p}^{T}$ is the weighting vector of the *p*-th pulse and $\zeta_{k,l}^{\prime }=\sqrt{E/M}\zeta_{k,l}$. Decompose the signal $y_{p,l}\left(t\right)$ by *M* matched filters. The filtered signal corresponding to the *m*-th transmitted waveform can be expressed as:

(41) $$\begin{array}{cc} {\bar{y}}_{p,l}\left(t\right) & =\int_{T_{p}}y_{p,l}\left(t\right)\phi_{m}^{*}\left(t\right)dt\\ & =H_{p}^{T}\sum_{k=1}^{K}\zeta_{k,l}^{\prime }b\left(\theta_{k}\right)a_{m}^{T}\left(\theta_{k}\right)+{\bar{z}}_{p,l}\left(m\right) \end{array}$$

where ${\left(\cdot \right)}^{*}$ is the conjugation operator and ${\bar{z}}_{p,l}\left(m\right)=\int_{T_{p}}H_{p}^{T}z_{p,l}\left(t\right)\phi_{m}^{*}\left(t\right)dt$ is the noise vector after filtering. Stacking *M* filtered signals in one row vector, we obtain the signal ${\bar{y}}_{p,l}=\left[{\bar{y}}_{p,l}\left(1\right),\ldots ,{\bar{y}}_{p,l}\left(M\right)\right]$. Assume that one data frame contains *N* pulses. The single channel MIMO signal of the *l*-th frame ${\bar{Y}}_{l}$ is:

(42) $${\bar{Y}}_{l}=\left[\begin{array}{c} {\bar{y}}_{1,l}\\ \vdots \\ {\bar{y}}_{N,l} \end{array}\right]=H^{T}\sum_{k=1}^{K}\zeta_{k,l}^{\prime }b\left(\theta_{k}\right)a^{T}\left(\theta_{k}\right)+{\bar{Z}}_{l}$$

where $H={\left[H_{1},\ldots ,H_{N}\right]}^{T}$ is an N×N Walsh–Hadamard matrix and ${\bar{Z}}_{l}$ is the noise matrix. Since $HH^{T}=NI_{N}$, the original MIMO signals ${\bar{X}}_{l}$ recovered from the single channel MIMO signals ${\bar{Y}}_{l}$ can be obtained by:

(43) $$\begin{array}{cc} {\bar{X}}_{l} & =\mathrm{vec}(\frac{1}{N}H{\bar{Y}}_{l})\\ & =\mathrm{vec}(\sum_{k=1}^{K}\zeta_{k,l}^{\prime }b\left(\theta_{k}\right)a^{T}\left(\theta_{k}\right)+\frac{1}{N}H{\bar{Z}}_{l})\\ & =\mathrm{vec}(\sum_{k=1}^{K}\zeta_{k,l}^{\prime }b\left(\theta_{k}\right)a^{T}\left(\theta_{k}\right))+{\tilde{Z}}_{l} \end{array}$$

where ${\tilde{Z}}_{l}$ is the observed noise.

Because both the transmit and receive arrays are sparse, the element positions of the recovered MIMO signal ${\bar{X}}_{l}$ should be slightly revised. For instance, if the configuration of the array is $P_{s}=\;\left\{1,4,14\right\}$ and $P_{r}=\left\{1,2,3,7\right\}$, the element index of the initially recovered MIMO signal ${\bar{X}}_{l}$ will be {1,2,3,7,4,5,6,10,14,15,16,20}. The revised signal ${\tilde{X}}_{l}$ can be obtained easily by rearranging the index of ${\bar{X}}_{l}$ in ascending order as {1,2,3,4,5,6,7,10,14,15,16,20}. To obtain the signals of the associated DCA of the single channel nested-MIMO array, the source covariance matrix R is calculated by:

(44) $$R=\frac{1}{L}\sum_{l=1}^{L}\mathrm{vec}({\tilde{X}}_{l})\mathrm{vec}({\tilde{X}}_{l}{)}^{H}$$

where ${\left(\cdot \right)}^{H}$ is the conjugation transpose operator. The vectorization of R represents the signals of the associated DCA of the proposed array according to Equation (8). The recovered signals will be used for direction-of-arrival (DOA) estimation, adaptive beamforming, and so on.

## 5. Simulation Results

**Figure 7:**
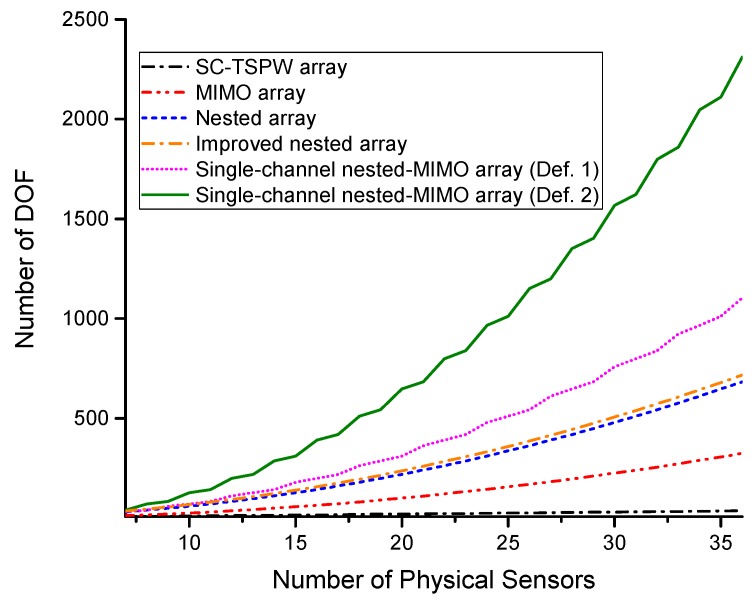
Comparison of the number of DOF versus the number of physical sensors.

The larger array aperture tends to have finer spatial resolution and smaller estimation error. For the restricted linear array [19], the number of DOF varies linearly with the aperture size. More DOF means more sources can be resolved. Therefore, in the first experiment, we compare the number of DOF provided by the single channel nested-MIMO array with that respectively provided by the conventional SC-TSPW array [11], MIMO array [34], nested array [16] and the improved nested array proposed in [41]. The total number of physical sensors of each array is consecutive integers from seven to 36. Specifically, the spacing between adjacent elements of the transmit array is N−1 times larger than that of the receive array to guarantee that the virtual array has no holes on the aperture. Figure 7 illustrates the comparison results. It is shown that the single channel nested-MIMO array constructed by Definition 2 provides the largest number of DOF among these arrays under the constraint of a fixed total number of sensors.

**Figure 8:**
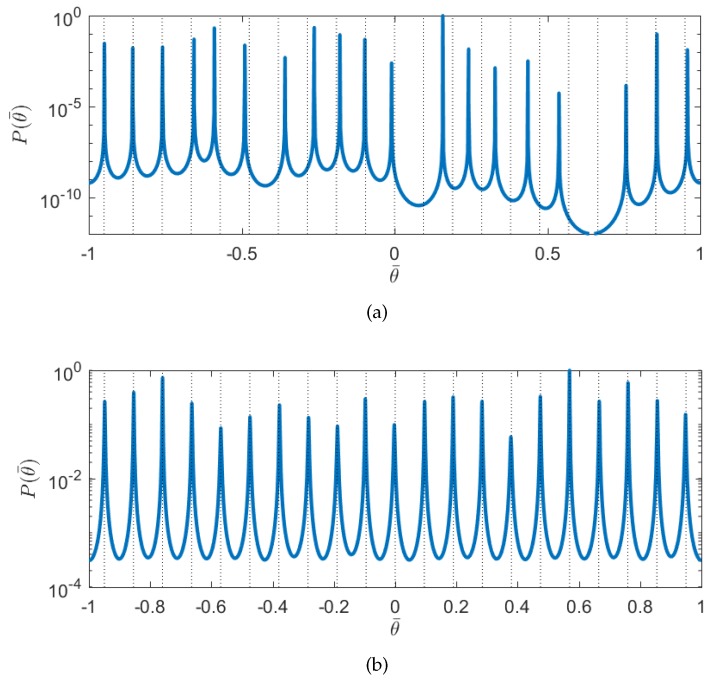
MUSIC spectra as a function of spatial frequencies. The true angles of the signal sources are depicted by the dash lines. (**a**) The spectrum of the nested array with eight physical sensors. (**b**) The spectrum of the single channel nested-MIMO array with four transmit antennas and four receive antennas.

The second experiment evaluates the performance of proposed array in terms of DOA estimation using the spatial smoothing multiple signal classification (SS-MUSIC) algorithm [16]. For comparison, the estimation result of a nested array is also presented. Each array has eight physical sensors. For the nested array, the number of DOF is 39. Since the SS-MUSIC can only exploit half number of DOF [16], the number of sources that can be resolved up by the nested array under this case is 19. The configuration of the single channel nested-MIMO array is $P_{s}=\left\{1,4,16,28\right\}$ and $P_{r}=\left\{1,2,3,9\right\}$. The number of DOF is 71, and the number of sources that can be resolved up is 35. Consider K=21 narrowband sources impinging on the array with equal power. The spatial frequencies $\bar{\theta }=\sin\left(\theta \right)$ are uniformly distributed in the range from −0.95 to 0.95. The noise is assumed to be Gaussian white noise. The signal to noise ratio (SNR) is 10 dB. The number of frames *L* is 300. Figure 8 illustrates the MUSIC spectra for the proposed array and a nested array, respectively. It can be seen that all of the targets have been identified correctly by the single channel nested-MIMO array, and the nested array failed to obtain the correct spectrum, as expected.

**Figure 9:**
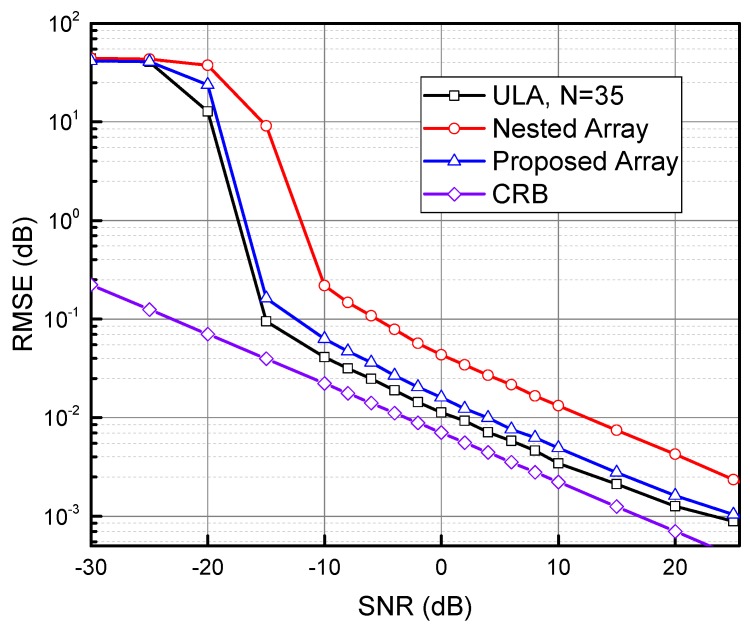
RMSE (in dB) versus SNR (for the source at $25^{\circ }$). Both the nested array and the proposed array have eight physical sensors.

Figure 9 shows the root mean square error (RMSE) of the angle estimates as a function of SNR, averaged over 2000 Monte Carlo simulations. Because the proposed array can identify 35 sources with the SS-MUSIC algorithm, we also consider the corresponding RMSE for conventional MUSIC applied to a 35-element ULA. The angle of signal source $\theta_{0}$ is $25^{\circ }$. The RMSE is defined as:

(45) $$\mathrm{RMSE}=\sqrt{\frac{1}{Q}\sum_{q=1}^{Q}{\left(\hat{\theta }-\theta_{0}\right)}^{2}}$$

where *Q* is the number of Monte Carlo simulations and $\hat{\theta }$ is the estimated angle. It can be seen that both the proposed array and the nested array perform worse than the conventional ULA with 35 elements. Because the Khatri–Rao product used to form the DCA is only an approximation when the number of samples is finite [16], however, the proposed array performs reasonably better than the nested array when both of them have the same number of physical sensors.

**Figure 10:**
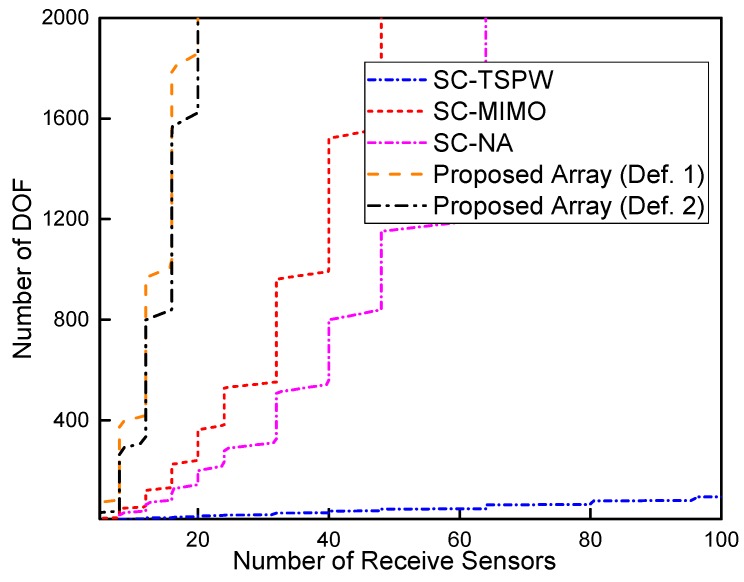
Number of receive sensors versus the number of DOF.

The single channel sampling time of the proposed array is evaluated in the third experiment. Because the sampling time of the single channel arrays scales linearly with the number of receive antennas, it suffices to compare the number of receive sensors rather than the single channel sampling time. For the purpose of comparison, we also show the numbers of receive sensors of the MIMO array and the nested array besides the SC-TSPW array, because the receive parts of these arrays can be naively modified with the single channel technology to reduce the cost. Figure 10 shows the number of receive sensors of the SC-TSPW array, the MIMO array with single receiver channel (SC-MIMO), the nested array with single receiver channel (SC-NA) and the proposed array. It can be observed that the proposed array requires the fewest number of receive sensors among those arrays. The number of receive sensors of the proposed array does not increase linearly with the number of DOF. However, it should be noted that the entire processing time of the single channel system is still longer than that of the multichannel system because the hardware cost saving is achieved at the cost of increased sampling time.

## 6. Conclusions

In this paper, we proposed a low-cost nested-MIMO array for large-scale wireless sensor applications. It enjoys the advantages of a single channel array, MIMO array and nested array. Not only are the hardware costs and the power consumption reduced, but the number of DOF is also effectively increased. Additionally, the single channel sampling time of the proposed array does not increase linearly with the number of DOF, which makes it possible to improve the real-time processing performance for large-scale wireless sensor applications. The effectiveness and superiority of the proposed array design strategy are verified by numerical results.

## Figures and Tables

**Table 1:** Sensor position of the virtual array ($N_{1}$ is even and $N_{2}$ is odd).

	$P_{t}$	1	j+2	5j+9	6j+10	10j+16	⋯	$4j^{2}+8j+4$
$P_{r}$								
1		1	j+2	5j+9	6j+10	10j+16	⋯	$4j^{2}+8j+4$
⋮		⋮	⋮	⋮	⋮	⋮	⋱	⋮
j+1		j+1	2j+2	6j+9	7j+10	11j+16	⋯	$4j^{2}+9j+4$
2j+3		2j+3	3j+4	7j+11	8j+12	12j+18	⋯	$4j^{2}+10j+6$
4j+6		4j+6	5j+7	9j+14	10j+15	14j+21	⋯	$4j^{2}+12j+9$

## Abbreviations

The following abbreviations are used in this manuscript:

TSPW	time-sequence-phase-weighting
MIMO	multiple-input multiple-output
DOF	degrees of freedom
CDMA	code division multiple access
DCA	difference co-array
NMRA	nested minimum redundancy array
SS-MUSIC	spatial smoothing multiple signal classification
RMSE	root mean square error
SNR	signal to noise ratio
SC-TSPW	single channel TSPW
SC-MIMO	single channel MIMO
SC-NA	single channel nested array
